# The Interaction between Coffee: Caffeine Consumption, UCP2 Gene Variation, and Adiposity in Adults—A Cross-Sectional Study

**DOI:** 10.1155/2019/9606054

**Published:** 2019-01-02

**Authors:** Harry Freitag Luglio Muhammad, Dian Caturini Sulistyoningrum, Emy Huriyati, Yi Yi Lee, Wan Abdul Manan Wan Muda

**Affiliations:** ^1^Department of Nutrition and Health, Faculty of Medicine, Public Health, Nursing, Universitas Gadjah Mada, Yogyakarta 55281, Indonesia; ^2^United Nation University, Kuala Lumpur 16150, Malaysia; ^3^Kyoto University, Kyoto 606-8501, Japan

## Abstract

**Background:**

Coffee is suggested as an alternative option for weight loss but the relationship between coffee consumption and adiposity in population-based studies is still controversial. Therefore, this study was aimed at evaluating the relationship between coffee intake and adiposity in adults and to test whether uncoupling protein 2 (UCP2) gene variation was able to affect this relationship.

**Methods:**

This was a cross-sectional study conducted in male and female adults living in the urban area of Yogyakarta, Indonesia. Adiposity was determined based on body weight, body mass index (BMI), percent body fat, and waist and hip circumference. Data on coffee consumption and other dietary components were collected using a semiquantitative food frequency questionnaire along with other caffeine-containing beverages such as tea, chocolate, and other beverages. The −866 G/A UCP2 gene variation was analyzed using polymerase chain reaction-restriction fragment length polymorphism. The correlation between coffee intake and adiposity was tested using linear regression test with adjustment for sex, age, energy intake, table sugar intake, and total caffeine intake.

**Results:**

In all subjects, coffee intake was inversely correlated with body weight (*β* = −0.122, *p*=0.028), BMI (*β* = −0.157, *p*=0.005), and body fat (*β* = −0.135, *p*=0.009). In subjects with AA + GA genotypes, coffee intake was inversely correlated with body weight (*β* = −0.155, *p*=0.027), BMI (*β* = −0.179, *p*=0.010), and body fat (*β* = −0.148, *p*=0.021). By contrast, in subjects with GG genotype, coffee intake was not correlated with body weight (*β* = −0.017, *p*=0.822), BMI (*β* = −0.068, *p*=0.377), and body fat (*β* = −0.047, *p*=0.504).

**Conclusion:**

We showed that coffee intake was negatively correlated with adiposity, and this was independent of total caffeine intake. Additionally, we showed that the −866 G/A UCP2 gene variation influences the relationship between coffee intake and adiposity.

## 1. Introduction

To date, caffeine is regarded as the most frequently consumed stimulant in the world due to increasing consumption of coffee, tea, cocoa, and ready-to-drink beverages [1, 2]. Coffee is the most highly concentrated source of natural caffeine, followed by tea and chocolate. It has been estimated that the average US adult consumes about 180 mg/day of caffeine while global coffee consumption reached 7 million tons per year [3, 4]. Coffee contains several biologically active compounds such as caffeine, polyphenols, and diterpenes. Caffeine is a derivate of the xanthine group, a natural alkaloid, that is also found in tea leaves and some other plants. Caffeine is metabolized in the liver and acts as an antagonist of adenosine receptors. Because adenosine receptors play a pivotal part in energy metabolism and are widely expressed in various tissues, this interaction might explain the physiological effect of caffeine [5]. In addition to caffeine, coffee also contains several other components such as polyphenols (caffeic acid and chlorogenic acid) and diterpenes (cafestol and kahweol) [5, 6].

Because of those bioactive peptides, coffee was suggested as an alternative option for weight loss and improvement of body composition [7]. However, the relationship between coffee consumption and adiposity in population-based studies is still controversial. It has been reported that coffee consumption was positively [8, 9], inversely [10–13], or not associated [14–16] with the risk of obesity.

The effect of caffeine intake on body weight has been investigated separately from coffee intake. The effect of caffeine intake on adiposity has been showed in an animal trial [17], but studies in humans showed that caffeine intake had no long-term effect on body weight [18, 19]. It has been suggested that the insignificant impact caffeine on body weight was due to development of insensitivity to chronic caffeine intake [20]. Additionally, individual genetic variation might also affect the long-term effect of coffee and caffeine intake on adiposity in humans [21].

In this study, we evaluated the interaction between coffee, caffeine intake, and uncoupling protein 2 (UCP2) gene variation on adiposity. This gene was selected because previous studies showed the direct effect of caffeine on UCP2 expression in vitro [22] and in vivo [23]. The UCP is presented in mitochondria and has an ability to regulate energy expenditure [24, 25]. This protein has several subtypes including UCP1, UCP2, and UCP3. UCP1 is expressed in white and brown adipose tissues, skeletal muscle, and pancreatic cells. UCP2 is expressed in the majority of tissue, while UCP3 is expressed mainly in skeletal muscle and brown adipose tissue [26, 27]. It was previously shown that UCP2 gene polymorphism was associated with obesity [28, 29]. However, it is unclear how UCP2 gene polymorphism was associated with obesity.

This study was aimed at evaluating the effect of coffee consumption on adiposity and whether this association was due to caffeine or independent of caffeine intake. Additionally, we are also interested at evaluating the interaction between UCP2 gene polymorphism and coffee intake on adiposity.

## 2. Materials and Methods

### 2.1. Subjects

This is a part of an Indonesian cohort that investigates the effect of lifestyle and genetic variation on metabolic syndrome in adults. This study was conducted in adult men and women between 19 and 56 years of age, living in the urban area of Yogyakarta, Indonesia. A total of 504 individuals were recruited from 5 subdistricts of the city area. Those areas were chosen based on population density: 3 subdistricts represented high-density population, and 2 subdistricts represented medium-density population. The inclusion criteria were permanent resident (at least 2 years) in the area and agreeing to become subject of this study by signing the informed consent. The exclusion criteria were diagnosis of chronic diseases such as diabetes, cardiovascular disease or cancer, pregnancy at the time the study was conducted, cigarette smoker or previous smoker, following a strict diet, and problems with walking or conducting physical activity in the last 6 months. Ethical clearance was obtained from the Medical and Health Research Ethics Committee (MHREC) Faculty of Medicine, Universitas Gadjah Mada, Indonesia (KE/FK/791/EC/2015). This study followed the ethical guidelines of the 1975 Declaration of Helsinki. We followed the methods of Luglio et al. [30].

### 2.2. Anthropometric Measures

Body weight and body fat were measured using a digital body mass scale (0.01 kg precision, Omron Karada Scan HBF-375, Osaka, Japan) while height was measured using a wall-mounted tape measure (0.1 cm precision, GEA Medical, Jakarta, Indonesia). BMI was calculated by dividing body weight (kg) by the square of height (m). Waist and hip circumference was measured using an unstretched measuring tape. All anthropometric measurements were done by trained personnel using calibrated instruments. Adiposity was determined based on body weight, BMI, body fat, and waist and hip circumference.

### 2.3. Dietary Intake

Data on dietary intake were collected using a semiquantitative food frequency questionnaire (SQ-FFQ), and the analysis was based on Indonesian Food Database and United States Department of Agriculture (USDA, 2017). Data of habitual consumption of food items that were collected using the SQ-FFQ were translated into daily intake including total energy, fat, carbohydrate, protein, fiber, sugar, coffee, and caffeine [31]. Data on caffeine intake and caffeine-containing foods and beverages were also collected from SQ-FFQ. In this study, daily caffeine intake was calculated based on intake of coffee, tea, chocolate, and caffeine-containing soda.

### 2.4. Physical Activity

The International Physical Activity Questionnaire (IPAQ) was used to evaluate subjects' physical activity [32]. This includes information on the intensity and duration of several activities. Those include work/job, transportation, house-related work and maintenance, recreation, exercise, and leisure time-related physical activities. The overall information on physical activity was then calculated as MET (metabolic equivalent of task) score, which represents the amount of energy used for a certain type of activity. All the questionnaires in this study have been translated, validated, and used before [33].

### 2.5. Genotype Analysis

From each participant, a 10 mL blood sample was collected in ethylenediaminetetraacetic acid- (EDTA-) containing tubes. After collection, blood plasma and buffy coat were separated by centrifugation. The DNA sample was isolated from buffy coat using a commercial DNA extraction kit (Favorgen, Taiwan). UCP2 −866 G/A genotyping was done using polymerase chain reaction-restriction fragment length polymorphism (PCR-RFLP) with forward primer: 5'-CAC GCT GCT TCT GCC AGG AC-3' and reverse primer: 5'-AGG CTC AGG AGA TGG ACCG-3'. PCR conditions are 8 minutes of denaturation in 95°C followed by 35 cycles of 95°C for 1 minute (denaturation), 55°C for 1 minute (annealing), 68°C for 1 minute (extension), and 72°C for 7 minutes (final extension). The PCR product was then digested using BST UI enzyme digestion. Restriction fragments were resolved on a 3% agarose gel. Subjects were divided into 2 groups AA + GA and GG genotypes.

### 2.6. Statistical Analysis

Statistical analysis was conducted using JASP (the University of Amsterdam, The Netherlands). Anthropometric measures, physical activity, dietary, and caffeine intake were compared between AA + GA and GG genotypes using independent *t*-tests. The correlation between caffeine, coffee intake, and adiposity measures in all subjects was analyzed in 3 models: Spearman correlation test (model I) and linear regression with correction for age, sex, energy, and sugar intake (model II). We also analyze the correlation between coffee intake and anthropometric measures using the linear regression test with correction for age, sex, energy, sugar, and total caffeine intake (model III). To measure the gene-diet interaction, separate correlation analysis was done based on UCP2 genotypes (AA + GA and GG groups). The correlation analysis was done in a Spearman correlation analysis and in a linear regression analysis with correction for age, sex, energy, sugar, and total caffeine intake. The analysis was significant when *p* < 0.05.

## 3. Results

This was a cross-sectional study conducted in male and female adults living in the city of Yogyakarta, Indonesia. Characteristics of all subjects and UCP2 genotype-specific groups are shown in Table 1. From 503 subjects who were initially involved in this study, only 455 of those were analyzed for UCP2 gene variation. In this study, we showed that gender was equally distributed (49% vs. 51% for male and female, respectively). There were no differences in age, anthropometric measures, physical activity, dietary intake, and caffeine intake between genotypes.

To measure the relationship between coffee, caffeine intake, and adiposity, we analyzed consumption of caffeine-containing foods and beverages. In this study, we showed that tea consumption contributes to the biggest proportion of daily caffeine intake (72.7%) in the population of this study. Coffee consumption only contributes to 22.9% of total caffeine intake, while chocolate and caffeine-containing soda contribute to 3.9% and 0.5% of total caffeine intake, respectively.

The correlation between coffee, caffeine intake, and adiposity was analyzed using the correlation test and linear regression test with correction for age, sex, total energy intake, and sugar intake (Table 2). Total caffeine intake was not associated with any parameters of adiposity in this study. Coffee intake was negatively correlated with body mass index and hip circumference, but when we corrected for age, sex, total energy intake, and sugar intake, the results were not significant anymore. The linear regression test was also conducted to analyze the correlation between coffee intake and adiposity with additional correction total caffeine intake. Interestingly, in this study, we showed that coffee intake was associated with body weight, BMI, and body fat after the additional adjustment. This showed that the effect of coffee intake on adiposity was independent of caffeine intake.

The interaction between UCP2 gene variation, coffee intake, and adiposity in Indonesian adults are shown in Table 3. To evaluate the gene-diet interaction, we separated subjects based on their genotypes, AA + GA genotypes and GG genotype. In subjects with AA or GA genotypes, coffee intake was inversely correlated with body weight, BMI, and body fat. By contrast, in subjects with GG genotype, the coffee intake was not correlated with any parameters of adiposity in the correlation analysis or multiple linear regression analysis with correction for age, sex, energy, sugar, and caffeine intake.

## 4. Discussion

The objective of this study was to evaluate the relationship between coffee intake and adiposity and to test whether UCP2 gene variation had interaction with coffee intake on adiposity. We showed that coffee intake was inversely associated with adiposity, and this correlation was independent of total caffeine intake. Subjects in this study were separated into 2 groups based on −866 G/A UCP2 gene variation: AA + GA and GG genotypes. We reported that in subjects with AA or GA genotypes, coffee intake was inversely correlated with adiposity, while this association was not seen in GG genotype.

Although the effect of chronic coffee and caffeine consumption on the prevention of weight gain has been shown in animal trials [34–36], the relationship between coffee intake and adiposity in human is somewhat controversial. A study conducted in 5,995 Korean women showed that coffee consumption was positively associated with BMI and waist circumference [8]. By contrast, a study conducted in Danish [37] and American [38] population showed that coffee intake had a small effect on protection against weight gain. In this study, we supported the previous findings that coffee consumption was negatively correlated with adiposity parameters.

We argued that the effect of coffee intake on adiposity was via regulation of energy expenditure. This notion was initially proven by Acheson et al. [39], who showed that coffee administration was associated with the increased metabolic rate in obese and normal weight individuals. This finding was then supported by a study conducted by Koot and Deurenberg [40], who also added that body temperature was also increased due to coffee administration. However, it is important to note that those metabolic responses to caffeine intake were analyzed in a short period of time and does not guarantee that this effect can persist in the long run. It has been suggested that humans have the ability to adapt a chronic caffeine intake, and this might be the reason for lack of significance between a long-term caffeine intake and adiposity [19, 20, 41].

We reported that caffeine intake was not correlated with any adiposity parameters. Additionally, we also showed that the correlation between coffee intake and adiposity became stronger when it was corrected for total caffeine intake. These two findings suggested an indication that there is another bioactive compound in coffee that plays an important role as an antiobesity compound than caffeine. This was supported by studies in the animal trial that showed that intervention with decaffeinated coffee was associated with prevention towards weight gain [42, 43]. Although caffeine has been associated with increased metabolic rate, it was also reported that decaffeinated coffee polyphenols content of the coffee was also associated with the increased metabolic rate in the animal trial. Murase et al. [42] showed that administration of coffee polyphenols for mice with a high-fat diet was proven to prevent weight gain, enhance metabolic rate, and increase expression of UCP2 mRNA in adipose tissue.

UCP2 is a protein expressed in mitochondria of large number of tissues such as muscle, adipose tissue, and internal organs and involved in regulation of energy metabolism. This was confirmed by Barbe et al. [44] who showed that adipose UCP2 mRNA was positively correlated with the resting metabolic rate. In addition, data from the genetic studies showed that UCP2 gene variation was associated with differences in energy expenditure [41, 45]. As a consequence, UCP2 gene expression has been associated with obesity, and this has been confirmed in humans and animals [46–48].

In this study, we reported that for those with GG genotype, coffee intake was not associated with adiposity, but for those with AA/GA genotypes, coffee intake was inversely correlated with adiposity. From this result, we speculated that subjects with GG genotype had no response to coffee intake because coffee intake had less ability to affect energy metabolism via UCP2 expression. It was previously reported that the −866 G/A variation of UCP2 gene was associated with UCP2 mRNA expression at the intraperitoneal adipose tissue. Subjects with G allele of −866 G/A UCP2 gene polymorphism had lower UCP2 mRNA expression compared to those with the A allele [49]. Because UCP2 plays an important part in energy metabolism [49, 50], consequent reduction of the expression might lead to lower energy expenditure, and this has been confirmed in humans. Kovacs et al. [51] reported that GG genotype of −866 G/A UCP2 gene was associated with lower energy expenditure compared to other genotypes.

The interaction between UCP2, coffee intake, and adiposity was significant even after correction for several factors such as sex, age, total energy intake, table sugar intake, and total caffeine intake. This showed that the effect was independent of those factors. Sex and age are known factors that are associated with body weight and adiposity. Women are more likely to have a higher percentage of fat even with lower body weight and lower waist circumference. Correction with energy and table sugar intake in this study is important because it was a custom in our population to drink coffee with additional sugar. The correction with caffeine showed that the effect might be due to other phytochemical components in the coffee which were not caffeine.

There were several limitations in this study. First, data on coffee intake were presented based on assumption that majority of Indonesian adults consumed instant coffee. This is because the majority of subjects in this study did not own a coffee filter machine and espresso maker at home. The usual coffee intake was a direct dilution of coffee powder or instant coffee powder. This is important because caffeine content of each processing is different, thus might affect the intake of caffeine and other bioactive compounds in the coffee. Second, the majority of the study participants drink coffee and tea with added sugar. Therefore, we conducted the correlation analysis with correction for table sugar intake. Because this is a cross-sectional analysis, the conclusion which was drawn in this study cannot represent the causal effect. To our knowledge, there is no study that investigates whether UCP2 gene variation affected the metabolism of coffee polyphenols. Third, data on coffee and caffeine intake were based on an SQ-FFQ. It has been reported that dietary intake data of SQ-FFQ on beverages such as water, tea, and coffee tended to be underestimated [31]. Thus, a careful consideration should be made to interpret the result from this study.

## 5. Conclusions

In conclusion, this study reported that coffee intake was inversely correlated with adiposity, and this relationship was independent of caffeine intake. Additionally, this study also reported that UCP2 gene variation affected the relationship between coffee intake and adiposity in humans. Thus, further studies on the potential of other bioactive compounds of coffee on body weight might be interesting to be investigated in the future. Because of the lack of responsiveness of GG genotypes on coffee intake, individuals with this genotype might not receive the benefit from coffee consumption on improvement of body composition. Therefore, further study is needed to evaluate which dietary alternative should be implemented for those with GG genotype.

## Figures and Tables

**Table 1:** Characteristics of subjects.

	All (*n*=455)	AA + GA (*n*=283)	GG (*n*=171)	*p* ^*∗*^
*Male/female*	223/232	142/141	81/90	
*Age*	41.6 ± 10.9	41.6 ± 10.0	41.5 ± 12.1	0.917
*Height*	158.1 ± 9.4	158.1 ± 9.4	158.1 ± 9.2	0.963
*Anthropometric measures*				
Body weight (kg)	62.7 ± 13.3	62.9 ± 13.4	62.4 ± 13.1	0.724
Body mass index (kg/m^2^)	25.1 ± 5.2	25.2 ± 5.5	25.0 ± 4.8	0.626
Body fat (kg)	17.7 ± 7.7	17.8 ± 7.9	17.6 ± 7.5	0.822
Waist circumference (cm)	86.6 ± 12.7	86.0 ± 12.6	87.5 ± 13.0	0.232
Hip circumference	94.2 ± 11.2	94.2 ± 11.5	94.3 ± 10.9	0.924
*Physical activity (METs-min/week)*	5769 ± 5911	5941 ± 6006	5489 ± 5761	0.433
*Dietary intake (per day)*				
Total energy intake (kcal)	2562 ± 1127	2530 ± 1129	2614 ± 1126	0.449
Protein (g)	80.0 ± 41.3	79.0 ± 42.3	81.6 ± 39.5	0.517
Fat (g)	66.7 ± 46.2	64.2 ± 42.8	70.6 ± 51.2	0.153
Carbohydrate (g)	410.7 ± 199.5	408.6 ± 198.4	414.2 ± 201.9	0.772
Fiber (g)	24.0 ± 13.4	23.3 ± 13.1	25.1 ± 13.8	0.168
Sugar (g)	26.7 ± 24.2	25.3 ± 23.6	28.9 ± 25.1	0.128
*Caffeine and its sources (per day)*				
Total caffeine (mg)	87.3 ± 91.9	85.2 ± 93.2	90.7 ± 89.7	0.540
Tea (ml)	276.2 ± 301.0	269.9 ± 312.5	286.6 ± 281.8	0.566
Coffee (ml)	35.5 ± 96.2	34.5 ± 89.6	37.1 ± 106.4	0.787
Chocolate (mg)	3.5 ± 9.9	4.09 ± 11.91	2.60 ± 5.20	0.121
Caffeine-containing soda (ml)	0.12 ± 1.53	0.15 ± 1.78	0.09 ± 1.00	0.685

^*∗*^Independent *t*-test of comparison between subjects AA + GA and GG genotypes of −866 G/A UCP2.

**Table 2:** The correlation between caffeine, coffee consumption, and adiposity.

	Model I				Model II^∧^				Model III^*∗*^	
	Caffeine		Coffee		Caffeine		Coffee		Coffee	
	r	*p*	r	*p*	*β*	*p*	*β*	*p*	*β*	*p*
Body weight (kg)	0.007	0.883	−0.018	0.703	0.093	0.070	−0.034	0.453	−0.122	0.028
Body mass index (kg/m^2^)	0.035	0.459	−0.094	0.044	0.077	0.135	−0.066	0.154	−0.157	0.005
Body fat (kg)	0.025	0.596	−0.083	0.077	0.083	0.081	−0.048	0.262	−0.135	0.009
Waist circumference (cm)	0.037	0.428	−0.050	0.285	0.079	0.117	−0.030	0.503	−0.105	0.055
Hip circumference (cm)	0.044	0.354	−0.098	0.036	0.093	0.064	−0.019	0.672	−0.099	0.069

^∧^Corrected for age, sex, total energy intake, and sugar intake; ^*∗*^corrected for age, sex, total energy intake, sugar intake, and total caffeine intake.

**Table 3:** The interaction between UCP2 gene variation, coffee consumption, and adiposity.

	AA + GA genotypes (*n*=283)				GG genotype (*n*=171)			
	*r* ^*∗*^	*p* ^*∗*^	*β* ^∧^	*p* ^∧^	*r* ^*∗*^	*p* ^*∗*^	*β* ^∧^	*p* ^∧^
Body weight (kg)	0.022	0.717	−0.155	0.027	−0.028	0.717	−0.017	0.822
Body mass index (kg/m^2^)	−0.074	0.214	−0.179	0.010	−0.092	0.231	−0.068	0.377
Body fat (kg)	−0.145	0.015	−0.148	0.021	−0.077	0.313	−0.047	0.504
Waist circumference (cm)	−0.062	0.302	−0.120	0.080	−0.051	0.510	−0.026	0.730
Hip circumference (cm)	−0.068	0.253	−0.096	0.158	−0.062	0.421	−0.039	0.602

^*∗*^Spearman correlation analysis; ^∧^linear regression analysis with correction for sex, age, total energy intake, table sugar intake, and total caffeine intake.

## Data Availability

The data used to support the findings of this study are available from the corresponding author upon request.
